# Development of the Conversational Health Literacy Assessment Tool for maternity care (CHAT-maternity-care): participatory action research

**DOI:** 10.1186/s12913-024-10612-0

**Published:** 2024-01-24

**Authors:** Evi M.E. Vlassak, Elina Miteniece, Judit K.J. Keulen, Marjolein Gravendeel, Irene Korstjens, Luc Budé, Marijke J.C. Hendrix, Marianne J. Nieuwenhuijze

**Affiliations:** 1 Research Centre for Midwifery Science, Zuyd University of Applied Sciences, Universiteitssingel 60, 6229 ER Maastricht, The Netherlands; 2 Maastricht University, Care and Public Health Research Institute (CAPHRI), Maastricht, The Netherlands

**Keywords:** Health literacy, Health Communication, Maternal-child nursing, Patient-Centred Care, Participatory Action Research

## Abstract

**Background:**

Limited health literacy in (expectant) parents is associated with adverse health outcomes. Maternity care providers often experience difficulties assessing (expectant) parents’ level of health literacy. The aim was to develop, evaluate, and iteratively adapt a conversational tool that supports maternity care providers in estimating (expectant) parents’ health literacy.

**Methods:**

In this participatory action research study, we developed a conversational tool for estimating the health literacy of (expectant) parents based on the Conversational Health Literacy Assessment Tool for general care, which in turn was based on the Health Literacy Questionnaire. We used a thorough iterative process including different maternity care providers, (expectant) parents, and a panel of experts. This expert panel comprised representatives from knowledge institutions, professional associations, and care providers with whom midwives and maternity care assistants work closely. Testing, evaluation and adjustment took place in consecutive rounds and was conducted in the Netherlands between 2019 and 2022.

**Results:**

The conversational tool ‘CHAT-maternity-care’ covers four key domains: (1) supportive relationship with care providers; (2) supportive relationship within parents’ personal network; (3) health information access and comprehension; (4) current health behaviour and health promotion. Each domain contains multiple example questions and example observations. Participants contributed to make the example questions and example observations accessible and usable for daily practice. The CHAT-maternity-care supports maternity care providers in estimating (expectant) parents’ health literacy during routine conversations with them, increased maternity care providers’ awareness of health literacy and helped them to identify where attention is necessary regarding (expectant) parents’ health literacy.

**Conclusions:**

The CHAT-maternity-care is a promising conversational tool to estimate (expectant) parents’ health literacy. It covers the relevant constructs of health literacy from both the Conversational Health Literacy Assessment Tool and Health Literacy Questionnaire, applied to maternity care. A preliminary evaluation of the use revealed positive feedback. Further testing and evaluation of the CHAT-maternity-care is required with a larger and more diverse population, including more (expectant) parents, to determine the effectiveness, perceived barriers, and perceived facilitators for implementation.

**Supplementary Information:**

The online version contains supplementary material available at 10.1186/s12913-024-10612-0.

## Introduction

The World Health Organization (WHO) formally defines health literacy (HL) as “The cognitive and social skills, which determine the motivation and ability of individuals to gain access to, understand and use information in ways to promote and maintain good health” [1]. Limited health literacy (LHL), therefore, is the inability or limited ability of individuals to gain access to, understand and use information in ways to promote and maintain good health. The WHO regards enhanced HL and improved responsiveness of care providers to HL needs as key determinants in reducing health inequalities [2]. LHL is associated with poorer health outcomes, increased healthcare costs and difficulties in communicating with care providers [3–7].

LHL rates differ substantially between European countries (25–72%), in the Netherlands around 25% of the population has LHL [8]. Still, 25% is a substantial part of the population. LHL is an issue that healthcare organisations and providers need to be aware of and respond to in order to provide equitable access to appropriate care [9].

LHL is associated with personal characteristics, such as low educational level and socio-economic status, but people with higher educational levels or higher socio-economic status may also have LHL [7, 8]. Women with LHL are often more challenging to reach and engage with than women with higher HL. This is primarily due to their limited understanding of pregnancy-related health information, coupled with a lack of awareness on accessing health services and difficulties in communicating health concerns and navigating the healthcare system. Additionally, care providers often lack awareness of their patients’ LHL, possess insufficient communication skills tailored for such patients, and have a limited knowledge of the unique challenges and needs specific to women with LHL. These issues are further intensified by differences in culturally determined habits surrounding care and communication problems [10, 11]. LHL is also associated with a higher rate of unplanned pregnancies [12], later initiation of prenatal care [12], higher smoking rates [13], lower prevalence of breastfeeding [14–16], lower adherence to prescribed medications [12, 16, 17], a greater chance of caesarean birth and adverse neonatal health outcomes [18]. Therefore, it is important for care providers to estimate the (expectant) parents’ HL in order to offer tailored care. However, care providers often have problems estimating their patients’ HL [19–22]. They often do not give it enough formal attention [22] and frequently overestimate their patients’ HL [20, 21].

In the Netherlands, more than 85% of pregnant women start care in a midwife-led maternity care practice [23] and almost all women receive postnatal care from both a midwife and a maternity care assistant at home during the first eight days after childbirth. These care providers play a key role in providing tailored maternity care to (expectant) parents. However, to provide tailored maternity care, a good understanding of (expectant) parents’ HL is necessary. The Health Literacy Questionnaire is a validated instrument to measure HL [24]. It consists of 44 questions in nine identified constructs of HL. During routine consultation hours, using the extensive Health Literacy Questionnaire faces limitations. These include incomplete responses if completed before provider contact, the provider’s time constraints to review 44 questions during consultations, and the questionnaire’s closed format, which limits in-depth exploration of HL issues, potentially hindering effective responses. This makes it unsuitable to use in daily care practice [25] and prompted the development of the Conversational Health Literacy Assessment Tool (CHAT). This conversational tool, which was developed in Australia, enables care providers to gather information about the context in which people manage their health, thereby estimating their HL. The nine constructs of the Health Literacy Questionnaire were reduced to five key domains (Box 1) [25]. Research suggests that the CHAT is a feasible and efficient tool for gaining insight into HL needs among individuals with varying socio-demographic characteristics and with different health status [26].

**Table:** 

Box 1: Domains of the CHAT [25] Domains of the CHAT	
Domain 1	Supportive professional relationships
Domain 2	Supportive personal relationships
Domain 3	Health information access and comprehension
Domain 4	Current health behaviours
Domain 5	Health promotion barriers and support

The CHAT seems helpful for maternity care providers to estimate the HL of (expectant) parents, but the implementation of an innovation is more effective and sustainable if the innovation is tailored to the target audience [27, 28]. Therefore, implementation of the CHAT within maternity care requires that the tool is tailored to the needs and daily practice of the care providers in maternity care.

The overall aim of this study is to develop, evaluate, and iteratively adapt a CHAT-based conversational tool specific for maternity care. This tool is not intended to be a scientific validated questionnaire for assessing HL in (expectant) parents. Rather, it is designed as a practical conversational instrument for use by maternity care providers to explore and estimate the (expectant) parents’ HL in different domains. This tool will provide examples of questions maternity care providers may ask within different domains of HL to estimate (expectant) parents’ HL. The overarching goal is that maternity care providers are able to explore the different constructs of HL with (expectant) parents.

## Methods

### Study design

This mixed-methods study is based on the participatory action research (PAR) approach. PAR is a research methodology based on co-creation between stakeholders and researchers [29]. This was considered the most appropriate approach for the development of the conversational tool due to its emphasis on inclusiveness and collaboration. In our study, PAR offers the space and platform to explore the aspects of HL that need attention in (expectant) parents in a participant-driven manner. This allows maternity care providers and (expectant) parents to explore their ideas and perspectives through cycles of action and reflection, which is essential to create and sustain change. PAR used in this study consists of an iterative cycle containing six stages: motivation, orientation, diagnosis, development, action, and evaluation [30]. The first stage “motivation”, was prompted by signals from maternity care providers that health information was not always well understood by (expectant) parents. The researchers connected this issue to the (expectant) parents’ HL and extent to which the maternity care providers tailor care to the specific needs of those with LHL. In order to provide tailored care to (expectant) parents with LHL, maternity care providers must first gain insight into the (expectant) parents’ HL.

**Fig. 1:**
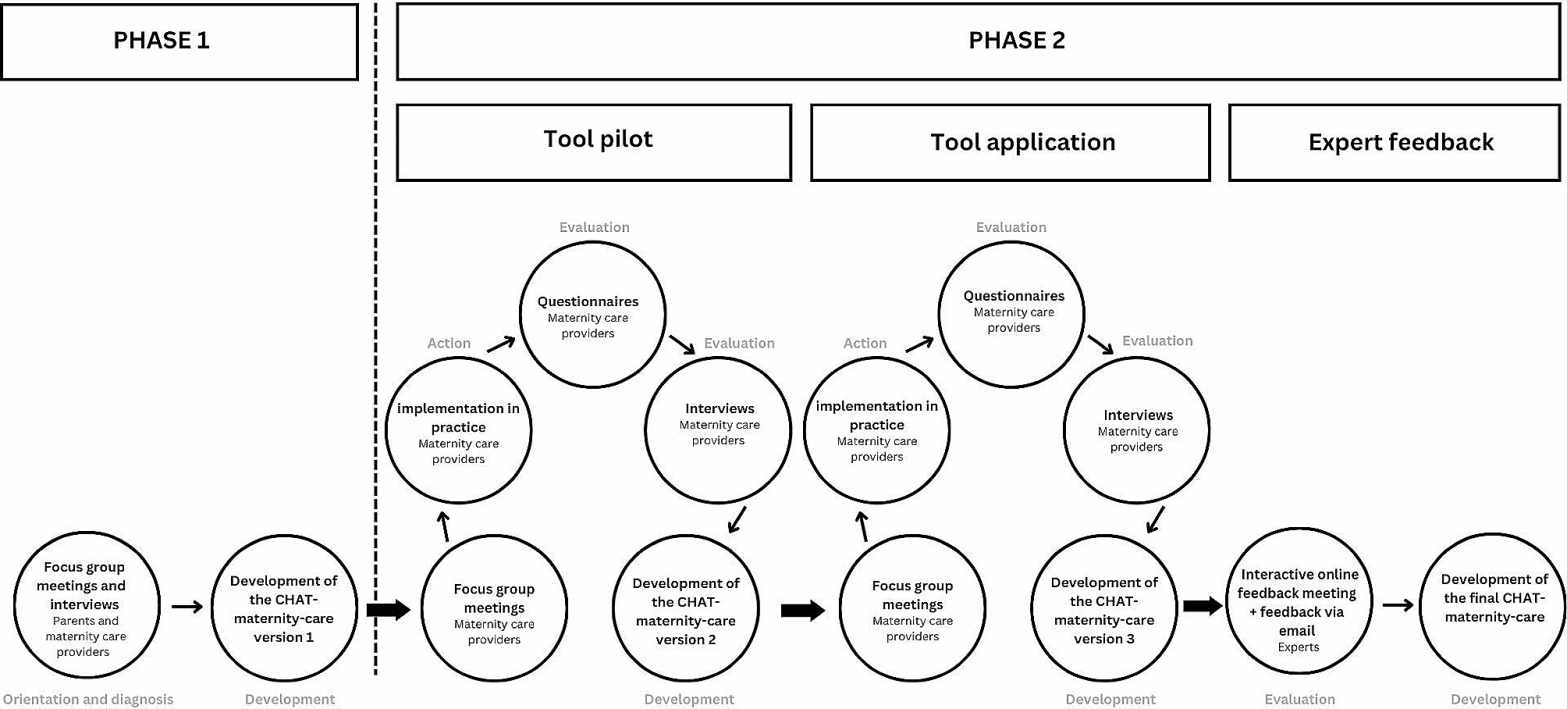
Flow diagram of the tool development including the stages of participatory action research

We developed the CHAT-maternity-care in two phases. Phase 1 contained three stages of PAR: orientation– diagnosis– development (Fig. 1). Phase 2 consisted of three iterations including four stages of PAR: action– evaluation– diagnose– development (Fig. 1).

### Information power

To determine the adequacy of our sample size, we applied the concept of ‘information power’ as described by Malterud et al. [31], considering several key factors:

- Study Aim: Our study’s aim was specific and focused, facilitating a high information power from each participant. This allowed us to gather rich, relevant data that was closely aligned with our research objectives.
- Sample Specificity: The participants in our study were chosen for their direct involvement and relevant experience in maternity care. This specificity significantly contributed to the information power of our sample, as participants provided in-depth and relevant insights.
- Theoretical Framework: Our research was informed by established theories in the fields of HL and maternity care, including the CHAT and the Health Literacy Questionnaire. This theoretical grounding facilitated a more nuanced understanding and interpretation of the data.
- Dialogue Quality: Our data collection process was carried out with a focus on high-quality dialogue. This was achieved through structured focus group meetings and interviews, ensuring in-depth and meaningful exchanges of information. Focus group meetings were conducted by teams of two, and interviews were led by experienced team members, further enhancing the quality of the dialogue.
- Analysis Strategy: The approach to analysing data in our study was methodical and targeted, focusing on specific aspects of maternity care and HL. This structured analysis strategy contributed to maximising the informational value of the data collected.

These considerations collectively informed our decision-making process regarding the sample size, ensuring that we gathered sufficient and rich data to meet our research objectives, while also complementing the concept of data saturation.

### Study participants and recruitment

Eligible study participants were primary care midwives, maternity care assistants, and women —and their partners— who were pregnant or gave birth between 2018 and 2020. All participants lived in the southeast of the Netherlands. In the rest of this article, we further refer to parents, meaning both parents and expectant parents. All study participants were recruited via email using purposive and snowball sampling. The email included an information sheet with information about the aim and relevance of this research, practical information, inclusion criteria, and contact details. Participants registered via email.

Furthermore, a panel of experts was consulted. This panel consisted of a professor in HL and patient participation, an obstetrician and participants representing the Koninklijke Nederlandse Organisatie van Verloskundigen [Royal Dutch Association of Midwives], the preventive Child and Youth Health Care Services, PHAROS (Dutch national centre of expertise on health disparities) and maternity care assistant organisations.

### Ethics

The study was assessed by the ethics board of Maastricht University Medical Centre. According to this ethics board the current research is not subject to the Medical Research Involving Human Subjects Act (WMO) and does not require ethical approval(number 2022–3283). All participants gave written informed consent to participate in the study. The consent form guaranteed participants full anonymity and confidentiality. Participants were free to decline participation or to withdraw at any time. Data was stored on a secured server, only accessible by members of the research team.

### Phase 1. Development of the first version of the CHAT-maternity-care

The aim of phase 1 was to collect information from maternity care providers and parents about their needs and views regarding HL to develop the first version of the conversational tool CHAT-maternity-care (stages of PAR: orientation– diagnose– development).

#### Data collection

Data collection was conducted by members of the research team (MG, IK, LB, MN). This took place in December 2019 in audio-recorded focus group meetings and interviews with parents, and in January 2020 in audio-recorded focus group meetings with maternity care providers. The topic guides for the focus group meetings and interviews were based on the five domains and ten questions of the original CHAT [25], which helped covering all relevant HL constructs (Appendix 1).

#### Data analysis

All recordings from the focus group meetings and interviews were transcribed verbatim. We re-listened the recordings and re-read the anonymized transcripts to facilitate deeper engagement with the data. Thereafter, the recordings were deleted. Subsequently, we coded and analysed the data using deductive content analysis in Nvivo 11. The domains of the CHAT were used as the initial framework for coding the data, other codes emerged from the data. Two members of the research team (MG, MN) categorised the codes into themes and subthemes. The analysed data were used to develop the first version of the conversational tool CHAT-maternity-care. A representative from PHAROS reviewed the CHAT-maternity-care and validated the readability and appropriateness of language used for people with LHL. Permission for using the acronym CHAT in combination with maternity care was granted by the authors of the original CHAT.

### Phase 2. Testing and evaluating of the CHAT-maternity-care

The aim of phase 2 was to test, evaluate, and adjust the CHAT-maternity-care (stages of PAR: action– evaluation– diagnosis– development). In an iterative process of three cycles, we created the final version of the CHAT-maternity-care.

#### Data collection

We collected data in three stages: tool pilot, tool application and expert feedback. During the tool pilot and tool application stage, we used focus group meetings, online questionnaires, and in-depth interviews with midwives and maternity care assistants to collect input on the content and usability of the CHAT-maternity-care. For efficiency, the testing and evaluation of the CHAT-maternity-care was first performed in a pilot group (tool pilot) from February until April 2022. After this, the research team further adjusted the CHAT-maternity-care, based on participants’ feedback and the original Health Literacy Questionnaire [24]. Subsequently, another round of testing and evaluation of the CHAT-maternity-care was performed (tool application) from May until July 2022. The expert feedback was provided in an online meeting and via email in September 2022.

During the tool pilot and tool application, members of the research team (EV, EM, JK) conducted eight online focus group meetings with midwives and maternity care assistants. The aim of these focus group meetings was to introduce maternity care providers to the concept of (limited) HL, to explain how to use the CHAT-maternity-care and to discuss and collect feedback on the CHAT-maternity-care. Maternity care providers were asked to use the example questions and example observations that were most suitable. During the focus group meetings field-notes were made to summarise care providers’ opinions on the face validity and applicability of the CHAT-maternity-care. After these focus group meetings, the maternity care providers tested the CHAT-maternity-care in their daily practice, and, subsequently, evaluated the use of CHAT-maternity-care through an online questionnaire. The questionnaire contained mostly open-ended, but also closed questions using a five-point Likert scale. The questions in the questionnaire were derived from instruments specifically designed to measure the determinants of innovations, namely MIDI [32], QQ10 [33], and Most Significant Change [34]. The topics of the questionnaire included the impact of the innovation’s characteristics, the individual adopting it, the socio-political context, and the organisation. The final question aimed to identify the most significant change resulting from the implementation of the innovation. To gain an in-depth insight in how care providers experienced the CHAT-maternity-care, one member of the research team (EV) also interviewed maternity care providers using an interview guide based on the results from the questionnaire (Appendices 2 and 3). We audio-recorded all interviews after consent from the participants.

Last, the panel of experts provided feedback in an interactive online meeting. Field-notes were made to summarise experts’ opinions on the face validity and applicability of the CHAT-maternity-care. Additional feedback from the experts was requested via e-mail.

#### Data analysis

The field-notes of the focus group meetings and the feedback of the experts were discussed by three members of the research team (EV, EM, JK). The questionnaires were analysed using descriptive statistics and through discussing the answers on the open-ended questions by two members of the research team (EV, EM). All recordings from the interviews were transcribed verbatim. Two members of the research team (EV, EM) re-listened to the recordings and re-read the anonymized transcripts to facilitate deeper engagement with the data. Subsequently, we coded and analysed the transcripts using inductive content analysis in Nvivo 11. The tool adjustments took place during an iterative process using the findings from the focus group meetings, questionnaires, in-depth interviews and feedback from the experts.

### Rigour and reflectivity

In this study, several strategies were used to ensure methodological rigour [35]. Data collection involved different methods such as focus group meetings, questionnaires, and in-depth interviews (methodological triangulation). Furthermore, at least two members of the research team analysed all data (investigator triangulation). All members of the research team had a background in maternity care or health sciences, and were experienced in qualitative and quantitative research. Regular meetings with the research team were held to discuss findings and adjustments to the CHAT-maternity-care. Data analysis and adjustment of the CHAT-maternity-care took place in an iterative process in which a new analysis also took the previous research findings into account. Data triangulation was secured by using various data sets during various phases of the study from different participant groups. As we primarily used a qualitative approach and there is no criteria list for PAR, we relied on the consolidated criteria for reporting qualitative research [36] to ensure comprehensive reporting of aspects of the research.

## Results

**Fig. 2:**
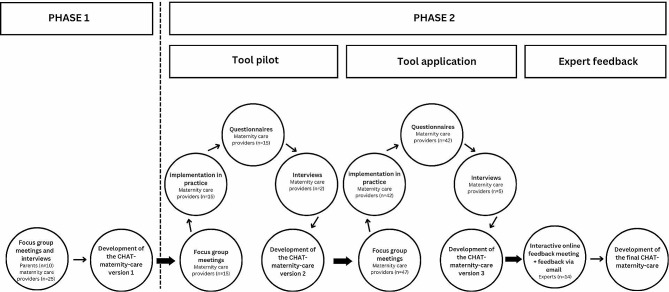
Flow diagram of the tool development including number of participants

A total of 72 maternity care providers, ten parents and 14 experts participated, divided over two phases. Phase 1 and phase 2 included different participants. However, the same participants have consistently participated in phase 2 (e.g. participants from the tool pilot stage also took part in the subsequent stage of tool application). The development of the first version of the CHAT-maternity-care took place in two focus group meetings with in total ten parents, five interviews with parents and four focus group meetings with 25 maternity care providers (Phase 1, Fig. 2). For the participants in phase 1 there are no participant characteristics available. The first version of the CHAT-maternity-care was pilot tested by 15 maternity care providers, nine midwives and six maternity care assistants (no participant characteristics available), followed by the development of the second version of the CHAT-maternity-care (Phase 2, Fig. 2). Feedback on this version was collected during the tool application stage from 47 maternity care providers in focus group meetings. Hereafter, five participants (three midwives and two maternity care assistants) withdrew, two due to time constraints and three without listing a reason. A total of 42 maternity care providers (17 midwives (age: 27–59 years, mean 40.9 years; working experience: 3–35 years, mean 15.7 years) and 25 maternity care assistants (age: 23–62 years, mean 43.4 years; working experience: 1–44 years, mean 16.3 years)) applied the second version of the CHAT-maternity-care in practice and provided their feedback (Phase 2, Fig. 2). With this feedback, we developed the third version of the CHAT-maternity-care. In the expert feedback stage 14 experts gave their feedback on the third version of the CHAT-maternity-care during an interactive expert meeting (*n* = 13) and/or by email (*n* = 2) (Phase 2, Fig. 2). Subsequently, the final version of CHAT-maternity-care was developed.

### Phase 1. Development of the first version of the CHAT-maternity-care

In the focus group meetings and interviews, both parents and maternity care providers emphasised the importance of discussing Domain 1 (Supportive professional relationships) and 2 (Supportive personal relationships) of the CHAT (Box 1). Parents highlighted the importance of care providers inquiring about which care providers they would consult during the perinatal period and, about the support of their family and how they perceive it. The parents mentioned that a supportive social network is important for them, and it typically consists of mothers, sisters, and other women of similar age or gestational age. The maternity care providers recommended discussing the supportive environment of parents during the perinatal period, including who they can rely on within their social network for advice and assistance.

Regarding Domain 3 (Health information access and comprehension), parents noted that it can be challenging to differentiate reliable information sources in an abundance of information and, therefore, suggested that maternity care providers should offer guidance in this. The maternity care providers expressed a sense of responsibility in providing parents with trustworthy information sources.

Regarding Domain 4 (Current health behaviours) and 5 (Health promotion barriers and support), both parents and maternity care providers highlighted the significance of discussing health-related knowledge, behaviour, and the associated challenges. Parents desired a personalised approach, while maternity care providers faced difficulties in discussing these domains as they wanted the parents to make their own health choices. The maternity care providers were hesitant to be judgmental during conversations, as it could potentially harm the relationship of trust with parents. The maternity care providers suggested discussing the facilitators and barriers in parents’ health behaviour, to more clearly identify the factors that could aid parents in adopting healthier behaviours and the obstacles that need to be overcome.

In general, maternity care providers indicated that observations often serve as a starting point to have a conversation about HL with parents and that they added to the conversation. Therefore, maternity care providers suggested to include example observations regarding each domain of the conversational tool. Consequently, examples of observations regarding the different domains of the CHAT-maternity care have been integrated into the CHAT-maternity-care from this point forward. The first version of the CHAT-maternity-care consisted of five domains including 24 example questions and 11 example observations which maternity care providers could choose from. A representative from PHAROS reviewed the CHAT-maternity-care and validated the readability and appropriateness of language used for people with LHL.

### Phase 2. Testing and evaluating of the CHAT-maternity-care

#### Tool pilot

For the CHAT-maternity-care in general, maternity care providers suggested that it was essential to formulate the example questions in the tool as openly and neutral as possible to allow parents formulating their own answers. According to the care providers, the way in which the parents formulate their answers gives additional insight into their HL. Furthermore, maternity care providers suggested to highlight keywords in the sentences, to use abbreviations, and to make a clearer distinction between Domain 1 (Supportive professional relationships) and 2 (Supportive personal relationships) to make the CHAT-maternity-care more user-friendly. For Domain 2 (Supportive personal relationships), maternity care providers suggested giving the partner more attention by adding an additional question about the availability of a supportive partner to talk to. For Domain 3 (Health information access and comprehension), they indicated that some questions were similar and could be merged. For Domain 4 (Current health behaviours) and 5 (Health promotion barriers and support), the maternity care providers pointed out that the example questions and example observations in both domains were similar. They suggested merging, because the domains were difficult to distinguish and to discuss separately.

All feedback was integrated, and the CHAT-maternity-care was adjusted respectively. The Domains 4 and 5 were merged, but a clear distinction between the two groups of questions were made: (1) current health behaviour and (2) health promotion. In addition, three researchers (EV, EM, JK) carefully reviewed and discussed the Health Literacy Questionnaire to identify any missing constructs that could support the estimation of parents’ HL. Consequently, we reformulated one example question and added three example questions and one example observation. This resulted in an adjusted version (version 2) of the CHAT-maternity-care with four domains that contained 21 example questions and 13 example observations.

#### Tool application

After using the CHAT-maternity-care in practice, maternity care providers indicated that it was an appropriate, clear, and helpful instrument to discuss HL with parents. They indicated that their awareness of HL had increased. Most maternity care providers pointed out that the CHAT-maternity-care supported starting a conversation on HL, gave guidance to explore HL, and facilitated a more extensive and complete conversation with the parents. This resulted in gaining better insight into the HL of the parents. Maternity care providers indicated that during the conversations using the CHAT-maternity-care, essential otherwise unknown information about the parents became apparent. However, some maternity care providers indicated that the CHAT-maternity-care was time-consuming. Other maternity care providers pointed out that talking about HL with parents is part of their work, and they felt confident enough to use the CHAT-maternity-care. Maternity care providers reported that parents responded well to the CHAT-maternity-care. Most maternity care providers said that they would recommend it to their colleagues and would continue using it themselves in the future.

Based on the feedback from maternity care providers during the tool application, minor adjustments were made to the questions and observations in all domains. A question within Domain 1 was deleted and several questions within Domains 2 and 4 were removed, adjusted, and merged. After the tool application stage, the CHAT-maternity-care contained four domains with 16 example questions and 12 example observations maternity care providers could choose from.

#### Expert feedback

The feedback from the panel of experts in the interactive online meeting and via email, led to some further small adjustments. Some questions were formulated differently to ensure the readability and appropriateness of language used for people with LHL. Furthermore, an observation was added that specifically focuses on low literacy. Feedback of the panel of experts resulted in the final version of the CHAT-maternity-care that contains four domains, including 18 example questions and 13 example observations (Table 1).

**Table 1:** final version of the CHAT-maternity-care

Domain	Question	Observations
1. Supportive relationship with care providers	Which care providers do you contact if you have a question about the pregnancy and the period thereafter?	Are other care providers involved?
	Do you know what questions to ask and which care provider to ask them to? Can you reach that care provider easily?	How do parents respond to care providers who visit them during the postpartum period?
	How does it make you feel to talk to that person about the questions or concerns you have?	Are the parents able to explain their problems/concerns well to you as a care provider?
2. Supportive relationship within parents’ personal network	With which people in your network (partner, family, friends, and neighbours) do you talk if you have questions about your pregnancy and the period thereafter?	Is someone else coming along to appointments? Is this always the same person?
	How does it make you feel to talk to that person/those persons?	After the baby is born, are there family, friends, and/or neighbours who can answer the parents’ health-related questions?
	Do you feel understood by that person/those persons?	Do the parents address each other’s health-related questions?
	Which person helps you best with health-related questions about you of your baby? How do they help you now? And how do you think they will help you in the future?	
3. Health information access and comprehension	Did you search/are you searching for information about the pregnancy and the period thereafter? Where did you find/are you finding that information?	What kind of questions do you receive from the parents?
	Can you find this information easily or is it difficult?	What information do the parents come to you with?
	What do you think of this information? - Do you know what information you can trust and which not? - Is this information difficult or easy to understand? - Is it too much, too little or just enough information?	What do parents do with the information they receive? Can they follow-up on instructions?
	How do you compare different information (sources)?	Are there signs that the parents have difficulties with writing or reading?
4. Current health behaviour and health promotion	How do you take good care of yourself and your baby?	Are the parents actively involved in their health?
	What do you do on a daily or weekly basis to stay healthy?	Do the parents ask for help?
	If you want to stay healthy during the period before and after the baby is born, what do you find easy and what difficult?	Are the parents able to take steps to behave healthily?
	Who or what helps you to live healthily during the pregnancy and the period thereafter? Who or what prevents this?	
	What do you want to do to live healthily?	

## Discussion

In this PAR study, we developed a conversational tool CHAT-maternity-care together with ten parents, 72 maternity care providers and a panel of 14 experts. The inclusion of ten parents, 72 maternity care providers, and 14 experts provided a comprehensive understanding of their experiences and perspectives. To support care providers with exploring and estimating the HL of parents, the CHAT-maternity-care offers 18 example questions and 13 example observations in four domains of HL. Domain 1 relates to the parent’s feelings of being understood and supported by care providers and their ability to actively engage with care providers and find their way in the healthcare system. Domain 2 relates to parent’s feelings of being understood and supported by their social network when faced with health-related issues. Domain 3 relates to finding, understanding, and appraising the necessary health information to manage their own health. Domain 4 relates to the parent’s current health behaviour and health promotion to actively improve their own health. Within these domains, the maternity care provider may choose which questions and observations to use, depending on the parents and their context. In our research, the use of the CHAT-maternity-care increased maternity care providers’ awareness of HL, it facilitated starting a conversation, gave guidance to explore the different aspects of HL, and resulted in conversations with the parents that were more comprehensive.

In our research, example observations were added to the CHAT-maternity-care because those example observations were an additional aid for maternity care providers in estimating the HL of parents and to start the conversation on HL with parents. Observations of behavioural patterns, also called informal methods, are the most common category of HL assessments [37]. However, using only observations as a way for assessment might lead to misjudging HL. This idea is supported by a study [38], which assessed 51 different methods of measuring HL, including both observations and self-reported data, and concluded that none of the methods assessed all dimensions of HL. Therefore, using only observations to determine HL also warrants caution.

Furthermore, besides the original CHAT, we also used the Health Literacy Questionnaire [24], the validated instrument for assessing HL on which the original CHAT is based. This was done to check whether the CHAT-maternity-care covers all relevant HL constructs, and if not to extend it, to further facilitate care providers in their conversations with parents. In the final version of the CHAT-maternity-care all nine constructs of the Health Literacy Questionnaire were integrated. This likely facilitates a comprehensive insight into the HL of parents. The conversational approach, as opposed to a questionnaire, can promote open communication and the development of stronger patient-care provider relationships, which might improve healthcare outcomes [39].

This study showed that the use of CHAT-maternity-care increased maternity care providers’ overall awareness of HL. This awareness of HL helps them to better adjust their information to the HL of the receiver of care [40]. Additionally, care providers who are aware of their role in adjusting care to HL, can increase the value that the care receivers place on health information [41]. Thereby, the CHAT-maternity-care could improve care for parents with LHL.

The CHAT-maternity-care was developed based on the iterative process of PAR, in which the end-users of the CHAT-maternity-care were involved in conceptualising and developing this tool. Iterative evaluation of an implementation process and real-time feedback-driven adjustments of an intervention are crucial for sustainable, context-appropriate intervention impact [42]. Thus, the iterative process increases the possibility of successfully implementing the CHAT-maternity-care in the Dutch maternity care system. Additionally, the feedback from the participants suggested that our tool the CHAT-maternity-care is easy to integrate in existing maternity care structures, because maternity care providers could selectively apply the example questions and example observations. Maternity care providers could also formulate their own questions within the concept of the domains as long as they kept a focus on all four domains to gain a complete overview of the HL of the parents. The utilisation of CHAT-maternity-care resulted in increased awareness of HL domains among maternity care providers, leading to their gradual internalisation and integration into practice. Although some care providers reported concerns that the adoption of CHAT-maternity-care could be time-consuming, we anticipated that prolonged use could establish HL considerations as a habitual component of care, ultimately requiring minimal time and effort investment. Further assessment should determine if and how the CHAT-maternity-care could be implemented in the Netherlands and beyond.

### Strengths and limitations

A limitation of this study is that maternity care providers who already felt closely involved in care for parents with a LHL might have been more willing to participate in study. Thus, selection bias cannot be excluded regarding the positive acceptance and usability of the tool. However, we expect the effect on the actual design of the tool to be minimal because the phrasing of the questions and observations does not strongly depend on the motivation of maternity care providers who participated. Furthermore, it is important to note that only ten parents participated in the development of the first draft of the CHAT-maternity-care, and no parents were involved in the second phase of the development. The recruitment of parents was intended in the second part, however, it proved impossible to recruit participants for the study. Therefore, the use of the CHAT-maternity-care in clinical practice was not assessed from the perspective of parents. Recruitment of parents was conducted through maternity care providers, which added two layers of complexity. For future research, a direct approach might be more effective. More maternity care providers than parents participated in the study, which might skew the results. Nevertheless, the researchers made an effort to consider the parents’ previous input throughout the entire development process. Moreover, it was a qualitative exploratory study, not quantitative, where the emphasis is on the frequency of similar findings. During recruitment, participants from diverse socioeconomic backgrounds were engaged. Additionally, PHAROS was involved to review the tool for clarity and comprehensibility. For research into the implementation of the CHAT-maternity-care, we strongly advise including (expectant) parents in the evaluation.

Strengths of this study are the methodological, investigator, and data triangulation. Research data was collected using focus group meetings, in-depth interviews, and questionnaires and was analysed with at least two members of the research team. Data analysis took place in an iterative cycle that allowed previously acquired data to be validated with data provided later. A further strength is the iterative process that actively involved stakeholders, and therefore, enabled progressive generation of knowledge and integrating necessary changes at every step. This provided a more complete, detailed, and balanced representation resulting in an increased external validity.

## Conclusion

After an iterative process of development using PAR, the CHAT-maternity-care is a promising conversational tool for maternity care providers to estimate parents’ HL. Our study suggests that CHAT-maternity-care increases maternity care providers’ awareness of HL and supports them in the conversation with the parents to estimate parents’ HL. This helps care providers to identify where attention is necessary regarding the parents’ HL and to adjust the care they provide to meet their needs. Tailoring care to parents’ HL will likely improve access to and use of care and potentially improve maternal and neonatal outcomes. Further testing and evaluation of the CHAT-maternity-care is required with a larger and more diverse population, including more (expectant) parents, to determine the effectiveness, perceived barriers, and perceived facilitators for implementation.

### Electronic supplementary material

Below is the link to the electronic supplementary material.

Supplementary Material 1

## Data Availability

The data used and analysed during the current study are available from the corresponding author on reasonable request.

## Abbreviations

CHAT: Conversational Health Literacy Assessment Tool
HL: Health literacy
LHL: Limited health literacy
PAR: Participatory Action Research

## References

[1] Nutbeam D (1998). Health Promotion Glossary.

[2] World Health Organization (2015). Optimizing health literacy: improving health and reducing health inequities.

[3] Schillinger D, Bindman A, Wang F, Stewart A, Piette J (2004). Functional health literacy and the quality of physician-patient communication among diabetes patients. Patient Educ Couns.

[4] Berkman ND, Sheridan SL, Donahue KE, Halpern DJ, Crotty K (2011). Low health literacy and health outcomes: an updated systematic review. Ann Intern Med.

[5] Cavanaugh KL, Wingard RL, Hakim RM, Eden S, Shintani A, Wallston KA, Huizinga MM, Elasy TA, Rothman RL, Ikizler TA (2010). Low health literacy associates with increased mortality in ESRD. J Am Soc Nephrol.

[6] Fransen MP, Stronks K, Essink-Bot ML, Gezondheidsvaardigheden. Stand Van Zaken, Laaggeletterdheid Te lijf edn. Centrum voor ethiek en gezondheid; 2011.

[7] Heijmans M, Zwikker H, Van der Heide I, Rademakers J, Kennisvraag. 2016: Zorg op maat. Hoe kunnen we de zorg beter laten aansluiten bij mensen met lage gezondheidsvaardigheden? In. Utrecht: NIVEL; 2016.

[8] Willems AEM, Heijmans M, Brabers AEM, Rademakers J. Gezondheidsvaardigheden in Nederland: factsheet cijfers 2021. In. Edited by NIVEL. Utrecht: NIVEL; 2022.

[9] Batterham RW, Hawkins M, Collins PA, Buchbinder R, Osborne RH (2016). Health literacy: applying current concepts to improve health services and reduce health inequalities. Public Health.

[10] College Perinatale Zorg. Zorgstandaard Integrale Geboortezorg Versie 1.1 In. Utrecht; 2016.

[11] Inspectie voor de gezondheidszorg. Mogelijkheden voor verbetering geboortezorg nog onvolledig benut. Samenvattend eindrapport van het inspectieonderzoek naar de invoering van het Advies van de Stuurgroep Zwangerschap en Geboorte. Utrecht; 2014.

[12] Endres LK, Sharp LK, Haney E, Dooley SL (2004). Health literacy and pregnancy preparedness in pregestational diabetes. Diabetes Care.

[13] Vila-Candel R, Navarro-Illana E, Mena-Tudela D, Pérez-Ros P, Castro-Sánchez E, Soriano-Vidal FJ, Quesada JA. Influence of Puerperal Health Literacy on Tobacco Use during pregnancy among Spanish women: a transversal study. Int J Environ Res Public Health 2020;17(8). 10.3390/ijerph17082910 PMC7216153 32340128

[14] Kaufman H, Skipper B, Small L, Terry T, McGrew M (2001). Effect of literacy on breast-feeding outcomes. South Med J.

[15] Yin HS, Sanders LM, Rothman RL, Shustak R, Eden SK, Shintani A, Cerra ME, Cruzatte EF, Perrin EM (2014). Parent health literacy and obesogenic feeding and physical activity-related infant care behaviors. J Pediatr.

[16] Poorman E, Gazmararian J, Elon L, Parker R (2014). Is health literacy related to health behaviors and cell phone usage patterns among the text4baby target population?. Archives of Public Health.

[17] Lupattelli A, Picinardi M, Einarson A, Nordeng H (2014). Health literacy and its association with perception of teratogenic risks and health behavior during pregnancy. Patient Educ Couns.

[18] Yee LM, Silver R, Haas DM, Parry S, Mercer BM, Wing DA, Reddy U, Saade GR, Simhan H, Grobman WA (2021). Association of Health Literacy among Nulliparous Individuals and maternal and neonatal outcomes. JAMA Netw Open.

[19] Ohl M, Harris A, Nurudtinova D, Cai X, Drohobyczer D, Overton ET (2010). Do brief screening questions or provider perception accurately identify persons with low health literacy in the HIV primary care setting?. AIDS Patient Care STDs.

[20] Dickens C, Lambert BL, Cromwell T, Piano MR (2013). Nurse overestimation of patients’ health literacy. J Health Communication.

[21] Storms H, Aertgeerts B, Vandenabeele F, Claes N (2019). General practitioners’ predictions of their own patients’ health literacy: a cross-sectional study in Belgium. BMJ Open.

[22] Creedy DK, Gamble J, Boorman R, Allen J (2021). Midwives’ self-reported knowledge and skills to assess and promote maternal health literacy: a national cross-sectional survey. Women Birth.

[23] Perined. Kerncijfers Nederlandse Geboortezorg 2020. In. Utrecht: Perined; 2020.

[24] Osborne RH, Batterham RW, Elsworth GR, Hawkins M, Buchbinder R (2013). The grounded psychometric development and initial validation of the health literacy questionnaire (HLQ). BMC Public Health.

[25] O’Hara J, Hawkins M, Batterham R, Dodson S, Osborne RH, Beauchamp A (2018). Conceptualisation and development of the Conversational Health literacy Assessment Tool (CHAT). BMC Health Serv Res.

[26] Jensen NH, Aaby A, Ryom K, Maindal HT (2021). A CHAT about health literacy - a qualitative feasibility study of the Conversational Health Literacy Assessment Tool (CHAT) in a Danish municipal healthcare centre. Scand J Caring Sci.

[27] Klein KJ, Sorra JS (1996). The challenge of innovation implementation. Acad Manage Rev.

[28] Fleiszer AR, Semenic SE, Ritchie JA, Richer MC, Denis JL (2015). The sustainability of healthcare innovations: a concept analysis. J Adv Nurs.

[29] Minkler M (2000). Using participatory Action Research to build Healthy communities. Public Health Rep.

[30] Migchelbrink F (2016). De Kern Van participatief actieonderzoek.

[31] Malterud K, Siersma VD, Guassora AD (2016). Sample size in qualitative interview studies: guided by Information Power. Qual Health Res.

[32] Fleuren MA, Paulussen TG, Van Dommelen P, Van Buuren S (2014). Towards a measurement instrument for determinants of innovations. Int J Qual Health Care.

[33] Moores KL, Jones GL, Radley SC (2012). Development of an instrument to measure face validity, feasibility and utility of patient questionnaire use during health care: the QQ-10. Int J Qual Health Care.

[34] Davies R. The ‘Most Significant Change’ (MSC) Technique: A Guide to Its Use; 2005.

[35] Korstjens I, Moser A, Series (2018). Practical guidance to qualitative research. Part 4: trustworthiness and publishing. Eur J Gen Pract.

[36] Tong A, Sainsbury P, Craig J (2007). Consolidated criteria for reporting qualitative research (COREQ): a 32-item checklist for interviews and focus groups. Int J Qual Health Care.

[37] Moore V (2012). Assessing health literacy. J Nurse Practitioners.

[38] Haun JN, Valerio MA, McCormack LA, Sørensen K, Paasche-Orlow MK (2014). Health literacy measurement: an inventory and descriptive summary of 51 instruments. J Health Communication.

[39] Stewart MA (1995). Effective physician-patient communication and health outcomes: a review. Can Med Assoc J.

[40] Güner MD, Ekmekci PE (2019). A Survey Study evaluating and comparing the Health Literacy Knowledge and communication skills used by Nurses and Physicians. Inquiry.

[41] Mårtensson L, Hensing G (2012). Health literacy - - a heterogeneous phenomenon: a literature review. Scand J Caring Sci.

[42] Luig T, Asselin J, Sharma AM, Campbell-Scherer DL (2018). Understanding implementation of Complex interventions in Primary Care teams. J Am Board Fam Med.

