# Supplementary material for: Development of the Conversational Health Literacy Assessment Tool for maternity care (CHAT-maternity-care): participatory action research
Source: BMC Health Serv Res. 2024 Jan 24;24:135. doi: 10.1186/s12913-024-10612-0 (PMC10809538; doi:10.1186/s12913-024-10612-0)
Supplement: Supplementary file 1 — Supplementary Material 1 [file 12913_2024_10612_MOESM1_ESM.docx]

## Appendix 1 Questions focus group meetings and interviews phase 1 maternity care providers and (expectant) parents

### **Questions focus group meetings and interviews (expectant) parents phase 1**

### Opening question

1. How did you experience the care from midwives, maternity care assistants, and the preventive Child and Youth Health Care Services during pregnancy, birth, and the first 2 years afterwards?
   1. What was your best experience and what was your worst? Why?

**[Define the term 'health literacy]**

### Health Literacy

1. During your pregnancy, postpartum period, and the first two years afterwards, were there situations where you felt less skilled in asking for, understanding, or applying the information you received?
   1. If yes and no elaboration: can you tell more about that/provide an example?

**[Research has been conducted on themes that healthcare providers can discuss when it comes to finding, understanding, and applying information. Five themes were identified in that research. We will now discuss these one by one through a series of questions.]**

### Domain 1: Supportive professional relationships

1. How do you view the relationship you had with healthcare providers during pregnancy, in the postpartum week, and the time thereafter?
   1. Do you look back on this positively or negatively? Why?
2. Did you receive any assistance in this? From whom? And if so, how was that assistance? Did you not receive assistance but would have liked to? In what aspect, from whom, and in what form would you have wanted this help?
3. How important is a supportive relationship with professionals during this period of your life?
4. Would you like to discuss this topic with healthcare providers?
   1. And at what moment?
   2. How can healthcare providers best approach this?
5. How have healthcare providers helped you adapt to pregnancy in relation to this topic, to live healthily, and to prepare for childbirth and parenthood?

### Domain 2: Supportive personal relationships

1. How do you view the personal relationships (with parents, partner, community) you had during pregnancy, in the postpartum week, and the time thereafter?
   1. Do you look back on this positively or negatively? Why?
2. Did you receive any assistance with this? From whom? And if so, how was that assistance? Did you not receive assistance but would have liked to? In what aspect, from whom, and in what form would you have wanted this help?
3. How important are supportive personal relationships during this period of your life?
4. Would you like to discuss this topic with healthcare providers?
   1. And at what moment?
   2. How can healthcare providers best approach this?
5. How have healthcare providers helped you adapt to pregnancy in relation to this topic, to live healthily, and to prepare for childbirth and parenthood?

### Domain 3: Health information access and comprehension

1. How do you view the information you had during pregnancy, in the postpartum week, and the time thereafter?
   1. Do you look back on this positively or negatively? Why?
2. Did you receive any assistance with this? From whom? And if so, how was that assistance? Did you not receive assistance but would have liked to? In what aspect, from whom, and in what form would you have wanted this help?
3. How important is access to health information and understanding it during this period of your life?
4. Would you like to discuss this topic with healthcare providers?
   1. And at what moment?
   2. How can healthcare providers best approach this?
5. How have healthcare providers helped you adapt to pregnancy in relation to this topic, to live healthily, and to prepare for childbirth and parenthood?

### Domain 4: Current health behaviours

1. Looking back on the first 1000 days of your child(ren)'s life, to what extent were you able to maintain healthy behaviours?
   1. Do you look back on this positively or negatively? Why?
2. Did you receive any assistance in this? From whom? And if so, how was that assistance? Did you not receive assistance but would have liked to? In what aspect, from whom, and in what form would you have wanted this help?
3. How important is healthy behaviour during the first 1000 days for you?
4. Would you like to discuss this topic with healthcare providers?
   1. And at what moment?
   2. How can healthcare providers best approach this?
5. How have healthcare providers helped you adapt to this topic during pregnancy, to live healthily, and to prepare for childbirth and parenthood?

### Domain 5: Health promotion barriers and support

1. Would you like to discuss this topic with healthcare providers?
   1. And at what moment?
   2. How can healthcare providers best approach this?
2. How have healthcare providers helped you adapt to this topic during pregnancy, to live healthily, and to prepare for childbirth and parenthood?

### Final questions

1. Are there any other domains where, according to you, healthcare providers should discuss with parents during the first 1000 days to optimally guide them in utilizing their health literacy?
   1. If so, which ones?
2. If you had to explain to a friend what we have discussed in this interview, what would you say?
3. Is there anything else you would like to tell or add?

### **Questions focus group meetings maternity care providers phase 1**

### Domain 1: Supportive professional relationships

**Original CHAT questions:**

Who do you usually see to help you look after your health?

How difficult is it for you to speak with [that provider] about your health?

1. How important is this topic in estimating someone's health literacy?
2. What questions would I ask about this topic to estimate someone’s health literacy?

### Domain 2: Supportive personal relationships

**Original CHAT questions:**

Aside from healthcare providers, who else do you talk with about your health?

How comfortable are you to ask [that person] for help if you need it?

1. How important is this topic in estimating someone's health literacy?
2. What questions would I ask about this topic to estimate someone’s health literacy?

### Domain 3: Health information access and comprehension

**Original CHAT questions:**

Where else do you get health information that you trust?

How difficult is it for you to understand information about your health?

1. How important is this topic in estimating of someone's health literacy?
2. What questions would I ask about this topic to estimate someone’s health literacy?

### Domain 4: Current health behaviours

**Original CHAT questions:**

What do you do to look after your health on a daily basis?

What do you do to look after your health on a weekly basis?

1. How important is this topic in estimating someone's health literacy?
2. What questions would I ask about this topic to estimate someone’s health literacy?

### Domain 5: Health promotion barriers and support

**Original CHAT questions:**

Thinking about the things you do to look after your health, what is difficult for you to keep doing on a regular basis?

Thinking about the things you do to look after your health, what is going well for you?

1. How important is this topic in estimating someone's health literacy?
2. What questions would I ask about this topic to estimate someone’s health literacy?

## Appendix 2 interview guide tool pilot

### Introduction and explanation of the research

Thank you for participating in this interview and the research project ZANGG. My name is Evi Vlassak, and I am one of the researchers involved in the project at the Maastricht Academy of Midwifery. I am a midwife myself. After using and evaluating the CHAT-maternity-care in a questionnaire, we now want to delve deeper into practical experiences with the CHAT-maternity-care. The results from both the questionnaires and this interview will be utilized to further develop and enhance the CHAT-maternity-care. All questions pertain to your own perspectives and experiences regarding the use of the CHAT-maternity-care. There are no right or wrong answers.

Participation in this interview is voluntary, and you may choose to stop at any time, even during the interview. All information will be anonymized and treated confidentially. The interview will be recorded. The results of this interview may also be included anonymously in a publication. The interview is expected to last approximately 30-60 minutes.

**[Start recording]**

### **[Today it is [date], and it is [time]. My name is [name]]**

### Determinants of the innovation

1. We received feedback from the questionnaires that the CHAT-maternity-care leads to more in-depth conversations. Do you think this also helps in identifying people with limited health literacy?
2. Was it clear to you how to use the CHAT-maternity-care? Why or why not?
3. Are the instructions clear?
4. Do you find the CHAT-maternity-care practical in the real-world setting? Why or why not?
   1. How can the questions or the way they are asked be improved?
   2. How can the appearance of the CHAT-maternity-care be improved?
   3. Do you still believe keywords could be helpful?
   4. It was mentioned in the questionnaires that it should be made shorter. We would like to implement this. Do you have any ideas on how we could do this?
   5. Would you recommend using the CHAT-maternity-care all at once, or would you suggest spreading the discussion of the domains across different moments?
5. From the questionnaires, we heard that using the CHAT-maternity-care took a lot of time. What would you change to overcome this implementation barrier?
6. Is there a domain overrepresented?
7. We would like to discuss the relevance of the questions for each domain:
   1. Domain 1:
      1. Are there questions that stand out to you, and why?
      2. Is there something relevant to limited health literacy that domain 1 does not ask about?
   2. Domain 2:
      1. Are there questions that stand out to you, and why?
      2. Is there something relevant to limited health literacy that domain 2 does not ask about?
   3. Domain 3:
      1. Do you think the last two points can be combined with the first point? How?
      2. Should we add a question about whether (expectant) parents are able to weigh information from different sources effectively?
      3. Are there questions that stand out to you, and why?
      4. Is there something relevant to limited health literacy that domain 3 does not ask about?
   4. Domain 4:
      1. Are there questions that stand out to you, and why?
      2. Is there something relevant to limited health literacy that domain 4 does not ask about?
   5. Domain 5:
      1. Are there questions that stand out to you, and why?
      2. Is there something relevant to limited health literacy that domain 5 does not ask about?

### Determinants of the user

1. How did you feel about using the CHAT-maternity-care?
2. Can you describe what you have learned by asking the questions from the CHAT-maternity-care?
3. Would you recommend the use of the CHAT-maternity-care to your colleagues? Why or why not?
   1. Do you think your colleagues would also use the conversation tool? Why?

### Determinants of the organisation

1. How is the CHAT-maternity-care incorporated into daily practice?
2. Are there practical issues that could hinder the implementation?

### Determinants of the socio-political context

1. How did (expectant) parents react to the CHAT-maternity-care?

### Most significant change

1. Can you mention what the most significant change is for you compared to before using the CHAT-maternity-care?
2. What is the most important thing to adjust in the CHAT-maternity-care?

**[Summarise]**

Is this correct? [waiting for an answer]

Do you have anything else to add or modify in this summary?

Then we want to express our sincere thanks for your contribution. In the coming months, we will make adjustments based on the questionnaires, focus groups, and feedback from experts.

## Appendix 3 interview guide tool application

### Introduction and explanation of the research

Thank you for participating in this interview and the research project ZANGG. My name is Evi Vlassak, and I am one of the researchers involved in the project at the Maastricht Academy of Midwifery. I am a midwife myself. After using and evaluating the CHAT-maternity-care in a questionnaire, we now want to delve deeper into practical experiences with the CHAT-maternity-care. The results from both the questionnaires and this interview will be utilized to further develop and enhance the CHAT-maternity-care. All questions pertain to your own perspectives and experiences regarding the use of the CHAT-maternity-care. There are no right or wrong answers.

Participation in this interview is voluntary, and you may choose to stop at any time, even during the interview. All information will be anonymized and treated confidentially. The interview will be recorded. The results of this interview may also be included anonymously in a publication. The interview is expected to last approximately 30-60 minutes.

**[Start recording]**

### **[Today it is [date], and it is [time]. My name is [name]]**

### Opening question

What, do you think, is the definition of health literacy?

### Determinants of the innovation

1. Why does the CHAT-maternity-care help (not) in communicating and identifying limited health literacy?
   1. If not ➔ How can we change this?
2. Is the CHAT-maternity-care too complicated to use?
   1. Why is this the case?
   2. What improvements can be made?
3. In the questionnaire some participants mentioned that the CHAT-maternity-care could be made shorter/more concise.
   1. Do you have any ideas on how we could achieve this?
4. The questionnaire responses indicated that time is a barrier to implementing the CHAT-maternity-care.
   1. What would you change to eliminate this implementation barrier?
   2. Would you recommend using the CHAT-maternity-care all at once, or would you suggest spreading the discussion of domains across different moments?

### Determinants of the user

1. Is the use of the CHAT-maternity-care uncomfortable for healthcare providers?
   1. Why?
   2. If yes ➔ What can we change to make this feeling less prominent?
2. Most participants feel that it is appropriate for their professional role to explore health literacy, but not the same percentage believes it is within their professional role to apply the CHAT-maternity-care.
   1. Why is this the case?
   2. Domain 1 is often indicated as not belonging to their role; why is that?
3. Most healthcare providers express their intention to continue using the CHAT-maternity-care in the future. However, not everyone plans to use the CHAT-maternity-care in the future.
   1. What do you need to continue using it in the future?

### Determinants of the organisation

Currently, there are no individuals within the organization/practice responsible for the implementation of the CHAT-maternity-care.

Would it be helpful if someone within the practice/organization were appointed to coordinate the implementation of the CHAT-maternity-care?

How would you implement the CHAT-maternity-care?

### Determinants of the socio-political context

1. Not everyone experiences support from colleagues.
   1. Why is this the case?
   2. Is support necessary to implement the CHAT-maternity-care?
   3. What can we change to promote support?
2. How did (expectant) parents react to the use of the CHAT-maternity-care?

### Most significant change

1. Can you indicate an order in the following concepts, from the most important change to the least important change (compared to before using the CHAT-maternity-care)?

Concepts derived from the questionnaire:

- It is supportive for the start of the conversation
- It provides a more complete and extensive conversation
- It creates time pressure
- It increases awareness among healthcare providers regarding the (expectant) parents’ health literacy
- It increases awareness among (expectant) parents regarding their own health literacy
- It provides structure within a conversation
- It contributes to better patient-provider contact and patient satisfaction

1. What have you missed after identifying limited health literacy in tailoring care for these (expectant) parents?

**[Summarise]**

Is this correct? [waiting for an answer]

Do you have anything else to add or modify in this summary?

Then we want to express our sincere thanks for your contribution. In the coming months, we will make adjustments based on the questionnaires, focus groups, and feedback from experts.
